# Affective and Attentional States When Running in a Virtual Reality Environment

**DOI:** 10.3390/sports6030071

**Published:** 2018-07-27

**Authors:** David L. Neumann, Robyn L. Moffitt

**Affiliations:** School of Applied Psychology, Griffith University, Queensland 4222, Australia; Robyn.Moffitt@vu.edu.au

**Keywords:** virtual reality, exercise, physical activity, sport, affect, attention

## Abstract

Engaging in physical exercise in a virtual reality (VR) environment has been reported to improve physical effort and affective states. However, these conclusions might be influenced by experimental design factors, such as comparing VR environments against a non-VR environment without actively controlling for the presence of visual input in non-VR conditions. The present study addressed this issue to examine affective and attentional states in a virtual running task. Participants (*n* = 40), completed a 21 min run on a treadmill at 70% of Vmax. One group of participants ran in a computer-generated VR environment that included other virtual runners while another group ran while viewing neutral images. Participants in both conditions showed a pattern of reduced positive affect and increased tension during the run with a return to high positive affect after the run. In the VR condition, higher levels of immersive tendencies and attention/absorption in the virtual environment were associated with more positive affect after the run. In addition, participants in the VR condition focused attention more on external task-relevant stimuli and less to internal states than participants in the neutral images condition. However, the neutral images condition produced less negative affect and more enjoyment after the run than the VR condition. The finding suggest that the effects of exercising in a VR environment will depend on individual difference factors (e.g., attention/absorption in the virtual world) but it may not always be better than distracting attention away from exercise-related cues.

## 1. Introduction

The application of virtual reality (VR) technology to enhance physical activity first became prominent in the 1990s. Improvements in technology, particularly graphical processing power, head-mounted displays, and high-speed internet, along with substantial industry investment, have led to a recent resurgence in interest [1]. Moreover, new developments have resulted in a blurring of the lines between VR, exergaming, and technology-facilitated training [2]. From a definitional sense, current VR applications refer to situations where an individual participates in a sport or physical exercise while in a computer-generated virtual environment that induces the feeling of presence, provides feedback, and enables interaction with the virtual world [2]. A key element of this definition is the notion that VR provokes a sense of presence in the virtual environment. Presence encompasses the factors of involvement and immersion [3]. The importance of presence makes VR different to exergaming, using video feedback, or even distraction techniques (e.g., watching a video) where presence is not required. Definitional issues are important when one considers the psychological processes through which VR might influence performance or affective states during physical exercise.

A recent systematic review on the use of VR in sport and exercise concluded that interactive VR has enhanced various performance, physiological, and psychological outcomes in both the short-term and long-term [4]. For example, researchers have reported that when compared to a control condition, engaging in physical activity in a VR environment has produced improved mood, reduced tiredness [5], and higher enjoyment [6,7]. However, the studies reviewed by Neumann et al. [4] were surprisingly lacking in design elements that could isolate one of the key definitional elements of VR, namely presence. For instance, of the 20 articles selected for review, half used experimental designs where all conditions involved some form of VR input. The lack of a non-VR comparison or control condition in these studies, thus, makes it difficult to determine the specific effects of experiencing presence in a VR environment when compared to a non-VR environment. The remaining studies did include some form of control condition. The typical control condition required that participants engage in the sport or exercise without any external stimulus being present (e.g., [5,6,7,8,9,10,11,12,13,14]). These manipulations have been referred to as a “blank environment” [13]. An example would be running on a treadmill that is facing a featureless wall.

While a comparison between exercise done in a VR environment and exercise done in a blank environment can inform what effects the addition of a VR protocol has, it is less informative regarding the mechanisms through which VR-based exercise exerts its effect. If a key feature of VR is to induce a feeling of presence in the virtual world, variations in the level of presence felt by individuals should be related to changes in affective states during VR-based exercise. One way to vary presence is through changing the methods by which the VR environment is presented. For example, presence will be larger when exercisers view the virtual world through a head-mounted display (HMD) that includes auditory stimuli and allows users to explore the world through head movements than when a simple computer monitor is used. However, a HMD is not always practical for exercising due to safety concerns and poor comfort when using exercise equipment [4]. Alternatively, individual differences in presence can be related to changes in affect during VR-based exercise. Presence can be measured using a rating scale completed after a VR task. Ijsselsteign et al. [15] used a virtual cycling task and reported correlations between subjectively measured presence and variables such as interest/enjoyment, perceived control, and pressure/tension. These findings support the role of presence in influencing affective states following VR-based exercise.

Another approach to examine the role of presence is to compare VR protocols with conditions that may more actively control for alternative explanations of the potential mechanism through which VR exerts its effects. Baños et al. [11], for example, suggested a virtual environment can be used to draw attention away from the perception of physical exertion, thereby improving affective states during exercise. The researchers conducted a study in which participants walked on a treadmill in a VR environment or walked in a blank environment. Significantly, their blank environment control condition was described as a traditional exercise situation in which participants focused their attention on physical sensations. The results supported this interpretation in that participants focused their attention on bodily sensations more in a blank environment than in a VR environment, although this difference interacted with the participant’s weight status (normal weight versus overweight). However, the investigators did not report statistical tests for the differences between the blank environment and VR environment separately for the two groups to determine if attentional focus was significantly different between the environments in both groups. Nevertheless, the results suggest that a virtual environment may serve as a mechanism by which to distract attention away from negative physical states that occur during exercise.

Mestre et al. [16] measured attentional focus using a visual analogue scale in which attention was defined by the end points of association (i.e., focusing on internal stimuli) and dissociation (focusing on external stimuli). Their procedure examined stationary cycling performance in a VR environment with a virtual coach pacer. Attentional focus was more internally focused in the blank environment condition than when cycling in a VR environment. Furthermore, these conditions produced more internally-focused attention than when cycling in the VR environment with a virtual coach. The authors suggested the VR promoted a distraction from the physical sensations associated with exercise intensity.

Based on the findings from prior research [11,16], it may be argued that attentional focus will be more likely to be directed to internal physical states when exercising in a blank environment than when exercising in a VR environment. However, prior research has not applied a systematic measure of attentional focus to provide a conclusive answer to this question. For example, the visual analogue scale used by Mestre et al. [16] used the end points of association and dissociation. This approach does not take into account that attention may also be allocated to internal or external cues. For example, the influential model of Stevinson and Biddle [17] uses a two-dimensional classification scheme of relevance (association versus dissociation) and direction (internal versus external). Internal association refers to cues related to the body or thought processes relevant to the task (e.g., body movements, self-talk, formation of strategies), whereas external association relates to task relevant cues in the environment (e.g., other competitors, the running track). Internal dissociation are thoughts that are unrelated to the task (e.g., daydreaming, planning a shopping trip), whereas external dissociation involves attending to environmental stimuli unrelated to the task (e.g., listening to music).

Other classification schemes for attentional focus exist. Brick et al. [18] reviewed existing conceptualisations for attentional focus in sport and exercise and proposed an extension to Stevinson and Biddle’s [17] two-dimensional scheme. In particular, for the associative categories, it was suggested that an external associative focus be conceptualised as a single category of outward monitoring. In contrast, it was suggested that an internal associative focus be divided into two types, namely internal sensory monitoring and active self-regulation. A similar approach was adopted by Wininger and Gieske [19] who described three internal associative conditions in their measure of attentional focus. To provide a more comprehensive assessment of attentional focus, the present study applied Wininger and Gieske’s [19] measurement tool which has a total of six categories: bodily sensations, task-relevant thoughts, self-talk, task-relevant external cues, task-irrelevant thoughts, and external distractions. The first three of these categories relate to an internal associative focus, whereas the remaining three relate to external association, internal dissociation, and external dissociation, respectively.

The present study also used a more active control condition than that afforded by employing a blank environment as used in prior research. Due to the prominently visual nature of virtual environments, it would be instructive to compare exercise in a VR environment with exercise while viewing a neutral visual stimulus (e.g., a video irrelevant to exercise or a sequence of neutral images). This type of active control condition would not induce a sense of presence in another world. However, it may be expected to encourage a dissociative attentional focus and a distraction away from bodily sensations while exercising. Thus, it would allow a dissection of the attentional mechanisms (i.e., presence and attentional focus) through which a VR environment produces its effects and whether these effects occur over and above the presentation of irrelevant stimuli.

The present study examined affective and attentional states during and following a treadmill running task. Participants completed the running task in a VR condition that simulated a virtual run through a park or in an active control condition that consisted of the presentation of neutral images. Participants ran at 70% of Vmax for 21 min, which was expected to be a challenging and vigorous task. Heart rate and perceived exertion were used to monitor exercise intensity and it was expected that both would increase throughout the task. Affective states were hypothesised to show a pattern of increasing activation and unpleasantness regardless of whether participants ran in a VR environment or when viewing neutral images. Affective states following the running task were also compared between conditions and different predictions were expected depending on how the conditions influenced attentional focus. If running in a VR environment reduces attention towards negative internal associative states (e.g., bodily sensations) more than running when viewing neutral images, it should produce higher positive affect and less negative affect. However, the opposite results for affective states are predicted if running when viewing neutral images reduces attention towards negative internal associative states more than running in a VR environment. The tendency to be immersed in virtual environments, in general, and the sense of presence induced by the VR running task, specifically, was also measured. It was expected that the tendency to be immersed and to feel presence would be associated with more positive and less negative affective states.

## 2. Method

### 2.1. Participants

Forty-six university students (21 male, 25 female) at Griffith University participated in exchange for partial course credit. Six participants were excluded from the study due to being unable to finish the running trial at the required intensity level. Participants were assigned to either a Virtual Reality group or Neutral Images group through matched assignment. Matching was based on age, Body Mass Index (BMI), and physical activity category level and metabolic equivalent (MET) as measured by the International Physical Activity Questionnaire-Short Form (IPAQ-SF; [20]). To provide greater statistical power for the correlational analyses involving presence in a virtual reality environment, more participants were allocated to the VR condition (*n* = 24 for Virtual Reality group and *n* = 16 for Neutral Images group). For this reason, a between groups design was used, otherwise the comparison between conditions would have been confounded by order effects if there were more participants in the virtual reality condition than the neutral images condition. The final sample consisted of 19 males and 21 females with a mean age of 24.50 years (*SD* = 9.12). As shown in Table 1, the two groups did not differ significantly in frequency of males and females, *χ*^2^ = (1) = 0.07, *p* = 0.80, age, *t* (38) = 0.07, *p* = 0.95, BMI, *t* (38) = 0.66, *p* = 0.51, IPAQ-SF category, *χ*^2^ = (2) = 0.14, *p* = 0.93, MET, *t* (38) = 0.21, *p* = 0.84, or Exercise Thought Questionnaire scores, *t* (38) = 0.60, *p* = 0.55.

### 2.2. Apparatus

The experiment was completed in a 2.5 m wide × 8 m long climate controlled room set with a light intensity of 10.1 lux. A model MW870UST BenQ data projector (BenQ, Taipei, Taiwan) was used to present visual stimuli at a resolution of 1920 × 1080 onto a white wall with a 2.5 m wide × 1.35 m high projection area. The VR environment was produced by the Netathlon^®^ 2XF running/skiing software (WebRacing, Madison, WI, USA). The VR environment did not use stereo vision. Ambient sounds from the VR environment (e.g., birds, wind, feet hitting the ground) were present and were played through speakers. The neutral images were produced using Powerpoint software (Microsoft Corporation, Redmond, WA, USA). Participants ran on a Marquee Fitness MT80 treadmill (Dyaco International Inc., Taipei, Taiwan) positioned 2 m away from the projected VR environment at its closest point. From this position, the VR environment or neutral images subtended a visual angle of 43.6° wide × 30.2° high from the point of view of the participant.

#### 2.2.1. Screening and Assessment Measures

*Pre-Exercise Screening System.* Stage 1 of the Pre-Exercise Screening System [21] was administered to participants to screen for pre-existing health conditions that might have precluded safe participation. The tool consisted of 23 *yes* or *no* questions that assesses current health concerns. No participants were excluded.

*Body Mass Index (BMI).* Participant’s height and weight were measured to calculate BMI. The formula used was BMI = weight (kg)/height (m^2^).

*International Physical Activity Questionnaire-Short Form (IPAQ-SF).* The IPAQ-SF [20] was used to classify participants into one of three physical activity categories (low, medium, and high) and to calculate metabolic equivalent (MET). The IPAQ-SF consists of seven items asking participants about the amount of time they spent engaging in vigorous and moderate exercise and how long they spent walking during the last seven days. The IPAQ-SF has been shown to positively correlate with physical fitness [22].

*Exercise Thoughts Questionnaire (ETQ).* The ETQ [23] measures the frequency of exercise avoidant thoughts using 25 statements about a person’s thoughts regarding exercise. Responses are given on a five-point scale ranging from 1 = *not at all* to 5 = *all of the time*. Scores are summed with higher scores indicating more frequent exercise avoidant thoughts. Internal consistency for the ETQ and other self-report questionnaires were examined using Cronbach’s alpha. An *α* greater than 0.7 was taken as evidence for sufficient internal consistency. The ETQ demonstrated high internal consistency in the current study (*α* = 0.94).

*The Immersive Tendencies Questionnaire.* The Immersive Tendencies Questionnaire (ITQ; [3]) measures how immersed participants can become in everyday activities and was used to examine how this trait might relate to the effects of running in the VR environment. The 18-item questionnaire is measured on a seven-point scale with the scale dependent on the question, for example 1 = *never* to *7 = often*, or 1 = *not at all* to *7* = *very well*. The ITQ has four subscales: focus, involvement, emotions, and games. The internal consistency for the ITQ was good in the current study (*α* = 0.77).

#### 2.2.2. In-Task Measures of Psychological States

*Perceived Exertion.* The Ratings of Perceived Exertion scale (RPE; [24]) was used to measure participant perceived exertion during the running task. The single-item scale ranged from 6 = *no exertion at all* to 20 = *maximal exertion*. The RPE has been positively correlated with heart rate (*r* = 0.80 to 0.90; [25]) and has shown high test-retest reliability (*r* ≥ 0.90; [26]).

*Affect.* Affect was measured using two dimensions of valence and arousal as proposed by the circumplex model of affect [27]. According to this model, each emotion can be mapped as a linear combination of the two basic neurophysiological systems of valence (pleasure-displeasure) and arousal (activation-deactivaton). Valence was measured by using the Feeling Scale (FS; [28]). The single-item measure is an 11-point bipolar scale that ranges from +5 = *very good* to −5 = *very bad*, with *neutral* at 0. The FS has shown convergent validity with the Self Assessment Manikin that measures affect valence (*r* = 0.51 to 0.88; [29]). Arousal was measured using the Felt Arousal Scale (FAS; [30]). The single-item scale ranges from 1 = *low arousal* to 6 = *high arousal*. The FAS has demonstrated convergent validity with other perceived activation measures [31]. 

#### 2.2.3. Post-Task Measures of Psychological States

*Physical Activity Affect Scale (PAAS).* The PAAS is a 12-item scale with four subscales: positive affect, tranquillity, fatigue, and negative affect [32]. Reponses are made on a 5-point scale ranging from 0 = *do not feel* to 4 = *feel very strongly* in response to whether a word describes how they feel about a physical activity that was just completed (e.g., “upbeat”, “calm”, “fatigued”). Internal consistency of the PAAS was good in the present study for positive affect (*α* = 0.82), tranquillity (*α* = 0.82), fatigue (*α* = 0.85), and negative affect (*α* = 0.76).

*Physical Activity Enjoyment Scale (PACES).* The PACES [33] measures enjoyment of physical activity. The scale consists of 16 first person statements relating to a physical activity that was just completed (e.g., “It’s a lot of fun”). Participants rate their level of agreement to each statement on a 5-point scale ranging from 1 = *totally disagree* to 5 = *totally agree*. The PACES internal consistency was high in the current study (*α* = 0.89).

*Intrinsic Motivation Inventory (IMI).* The Post-Experimental IMI [34] measured participant motivation during the exercise task. The IMI contains seven possible subscales although the following two subscales were used: effort/importance and felt pressure/tension. From this, the scale contained 10 first person statements (e.g., “I tried very hard in this activity”) that participants provided a rating from 1 = *not true at all* to 7 = *very true*. In the current study the IMI demonstrated good internal consistency for effort/importance (*α* = 0.84), and felt pressure/tension (*α* = 0.73).

*Measure of Attentional Focus (MAF).* The MAF [19] was used to identify participant’s attentional states during the task according to the association/dissociation and internal/external dimensions proposed by Stevinson and Biddle [17]. Participants were asked to report the percentage of time they spent thinking about six different categories. When framed within the two dimensional model of attentional focus of Stevinson and Biddle [17], three categories related to internal association (bodily sensations, task-relevant thoughts, self-talk), one category related to external association (task-relevant external cues), one category related to internal dissociation (task-irrelevant thoughts), and one category related to external dissociation (external distractions). The percentage given to all six categories were required to sum to 100%.

*Reality Judgement and Presence Questionnaire (RJPQ).* The RJPQ [35] assessed participant’s perception of the realism of the VR environment. The 16-item questionnaire was measured on a 10-point scale ranging from 0 (*not at all*) to 10 (*absolutely*). The RJQ has three subscales: reality judgement (*α* = 0.91), internal/external correspondence (*α* = 0.70), and attention/absorption (*α* = 0.58). The total scale also showed good internal consistency (*α* = 0.91).

### 2.3. Procedure

Following informed consent, participants completed the Pre-Exercise Screening System, reported their gender and age, and height and weight measurements were taken. The Vmax test was next completed as an indicator of participant running ability and has shown to be an equivalent or superior predictor of running performance than other test measures (e.g., VO_2_max; [36]). Participants initially ran on the treadmill at a low intensity pace to warm up and for familiarisation. Next, participants started running at a pace of 8 km/h and held this for 1 min. The pace was increased by 1 km/h and participants were asked to hold that pace for 1 min. This was continued until volitional exhaustion required the participant to stop running. The speed that was maintained for the final full 1 min was recorded as the participant’s maximum velocity. During the Vmax test, all information on the treadmill display (e.g., speed, distance, incline setting) was covered. The mean final speed in the Vmax test for the Virtual Reality group (*M* = 12.52 km/h, *SD* = 2.67) and Neutral Images group (*M* = 12.82 km/h, *SD* = 2.50) did not differ significantly, *t* (37) = 0.36, *p* = 0.72.

After the Vmax test, participants were provided with a rest period during which time they completed the IPAQ and ETQ. Participants were then allocated to either the Virtual Reality group or Neutral Images group and given instructions for the 21-min running trial. Participants in the Virtual Reality group were asked to run for the next 21 min whilst viewing a virtual environment on the screen in front of them. On the screen they saw themselves as an avatar running in a VR environment that was like “running through a park”. They were advised that there would be other virtual runners in the environment with them and that their view would change during the trial (for example, first person or third person). The trial began with a third person view. The view changed so that the trial began in third person view for 2 min, then switched to first person view for 2 min, and then switched to a third period view in which the perspective rotated around the participant’s avatar for 30 s, with this order repeated until the end of the trial. Participants were instructed that they did not need to do anything in response to the change in view and that they were just features of the software. The views were changed based on pilot testing which indicated that the running task was more engaging when the views changed. In addition, participants were not given any specific instructions in relation to the other avatars. The software was set to have eight other runners being present, with some avatars running at a faster pace than the participant’s pace, some at a slower pace than the participant’s pace, and some set to run at varying pace that was on average the same as the participant’s pace. Participants were also given instructions on how to make ratings for exertion, feeling, and arousal.

The instructions remained the same in the Neutral Images group with the exception that participants were instructed they would be running for the next 21 min whilst viewing pictures of objects on the screen. A sequence of 44 images were created by selecting 22 images that were rated as low in arousal and neutral in valence (e.g., a clock, basket, whistle, buttons) from the International Affective Picture System [37]. Image numbers were 7000, 7001, 7009, 7010, 7012, 7018, 7021, 7036, 7041, 7052, 7055, 7056, 7080, 7090, 7131, 7160, 7161, 7179, 7185, 7211, 7233, and 7547. The images were presented for 30 s each in random order with the restriction that the same image could not be presented more than twice across the entire running trial.

Following the instructions, participants completed the 21 min running trial in which the treadmill speed was set at 70% of their Vmax. Ratings of exertion, feeling, and arousal were requested from participants 5 min prior the task and then once starting the task at 1 min, 6 min, 11 min, 16 min, 21 min, and 5 min after completing the trial. In the time interval between the pre-task rating and commencing the running task, preparations for the measurement of heart rate were made. Heart rate was measured through electrocardiogram (ECG) recordings. Disposable surface mounted Ag/AgCl electrodes (Ambu T-sensor, Ballerup, Denmark) were attached to the manubrium and xiphoid process of the chest region, with the ground electrode placed over the lower right rib bone. The signal was acquired via an ADInstruments PowerLab 8/35 (ADInstruments, Dunedin, New Zealand) in conjuction with a FE132 BioAmp at a sampling rate of 1000 Hz and 10 Hz to 500 Hz bandpass filter. The resulting ECG signal was scored using the ADInstruments LabChart Pro ECG human detection algorithm to derive mean heart rate across each 1 min interval of the 21 min trial.

At the end of the running task, participants completed the post-task measures PAAS, PACES, IMI, and MAF. Participants were asked to complete these scales in respect to the overall task (i.e., not specifically how they felt at that point in time or right at the end of the running task, but across the entire task as a whole). Participants in the VR group were also asked to complete the RJPQ. At the appropriate time, participants were also to give their 5 min post-task ratings of RPE, FS, and FAS in relation to how they felt at that point in time. Participants were debriefed at the completion of the experiment.

### 2.4. Statistical Analyses

The primary independent variable was Group with two levels (Virtual Reality, Neutral Images). An additional independent variable of Time was used for the dependent measures in which there were repeated measurements prior to, during, and/or following the running trial. Time had five levels for RPE (1, 6, 11, 16, 21 min), seven levels for FS and FAS (pre5, 1, 6, 11, 16, 21, and post-5 min), and 21 levels for heart rate (1 to 21 min in 1 min intervals). In addition, the percentage allocations reported in the MAF were categorised according to the two dimensional classification scheme of Stevinson and Biddle [17] to create the independent variables of direction (internal, external) and relevance (association, dissociation). Analyses were also conducted to examine the three levels of the internal association categories on the MAF (bodily sensations, task-relevant thoughts, and self-talk). Following screening and checking of assumptions, comparisons between groups were made with *t* tests (for post-task measures) or mixed models ANOVAs (for within-task measures). For the *t* tests, adjusted degrees of freedom were used when Levene’s test indicated violation of the equal variances assumption. For the ANOVAs, Huynh-Feldt adjusted degrees of freedom were used for violations of the sphericity assumption. The relationships between measures were examined using bivariate Pearson correlations. Statistical significance was assessed against a Type I error rate of 0.05 for all analyses.

## 3. Results

### 3.1. Physical and Perceived Exertion during the Running Trial

Participants completed the 21 min running trial at 70% of the final speed reached in the Vmax assessment. This mean speed in the VR group (*M* = 8.66, *SD* = 1.32) and Neutral Images group (*M* = 9.00, *SD* = 1.71) corresponded to a moderate to vigorous run [38]. As shown in Table 2, the RPE values supported this interpretation. According to recommended physical activity intensity terminology [38], participants were engaging in light activity in the first minute of the trial and intensity steadily increased until completion. The physical activity was moderate by 6 min and reached a vigorous level by 16 min into the trial. A 2 × 5 (Group × Time) ANOVA confirmed the increase in RPE across the trial with a main effect of Time, *F* (2.26, 83.63) = 77.66, *p* < 0.001, η_p_^2^ = 0.68. Post hoc analyses employing *t* tests with Bonferroni corrections (α′ = 0.0125) were conducted to examine the differences across the successive time periods. RPE increased significantly from 1 to 6 min, from 6 to 11 min, from 11 to 16 min, and from 16 to 21 min, all *t*s > 3.34, *p* < 0.002, *d* > 0.53. However, the groups did not differ in the change in RPE over the trial or in overall RPE as shown by no significant Group × Time interaction or main effect of Group, both *F* < 1.20, *p* > 0.31.

**Figure 1:**
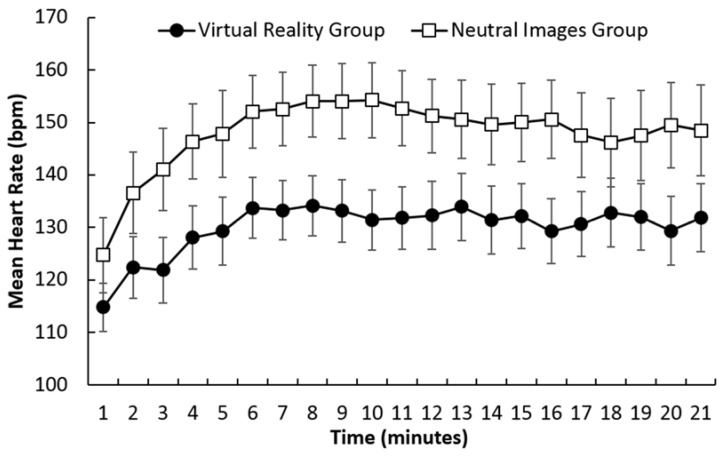
Mean heart rate for each 1 min time period across the 21 min running trial in the Virtual Reality group and the Neutral Images group.

As shown in Figure 1, mean heart rate increased from min 1 through to min 7, after which it remained relatively stable. A 2 × 21 (Group × Time) ANOVA confirmed the increase in heart rate across the trial with a main effect of Time, *F* (2.87, 103.51) = 8.99, *p* < 0.001, η_p_^2^ = 0.20. Bonferroni corrected *t* tests (α′ = 0.0025) were conducted to examine the differences between successive minutes (min 1 versus min 2, min 2 versus min 3, etc.). Heart rate increased significantly from 1 to 2 min, from 3 to 4 min, and from 5 to 6 min, all *t*s > 3.47, *p* < 0.001, *d* > 0.54. All other comparisons were not significant, all *t*s < 1.42, *p* > 0.16. There also appeared to be a difference between groups, with heart rate tending to be higher in the Neutral Images group than in the Virtual Reality group. The main effect for Group, *F* (1, 36) = 3.92, *p* = 0.054, η_p_^2^ = 0.09, approached significance and the Group × Time interaction, *F* (2.87, 103.51) = 0.51, *p* = 0.67, η_p_^2^ = 0.01, was not significant. In summary, the measures of physical and perceived exertion during the trial indicated that exertion increased across the trial, reaching a moderate to vigorous level after 6 min.

### 3.2. Psychological States during the Running Trial

**Figure 2:**
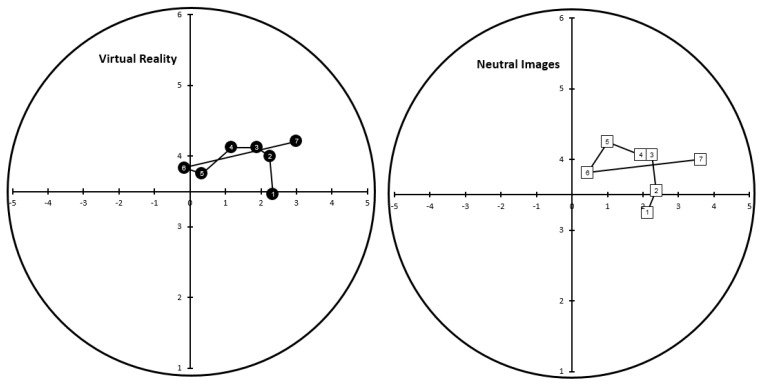
Circumplex space showing the mean ratings for the feeling scale (X-axis) and arousal scale (Y-axis) across the successive time periods (1 = pre-task, 2 = 1 min, 3 = 6 min, 4 = 11 min, 5 = 16 min, 6 = 21 min, 7 = post-task) in the Virtual Reality group (**left**) panel and Neutral Images group (**right**) panel.

Mean ratings for the FS and FAS were mapped onto the circumplex space and are shown in Figure 2. As can be seen, participants reported a moderate level of positive valence prior to and during the first 6 min of the trial. During this time felt arousal increased from a neutral to a moderately high level. Over the course of the remainder of the trial, feeling states became increasingly negative and arousal increased. Arousal decreased immediately after the trial and there was a rebound towards increased positive feeling states 5 min after trial completion. This pattern in feeling states across the running trial was similar for the two groups, although there was some evidence of higher arousal and more negative valence in the Virtual Reality group than in the Neutral Images group.

Separate 2 × 7 (group × time) ANOVA did not yield any significant effects involving the group factor for either FS, both *F*s < 0.74, *p* > 0.49, or FAS, both *F*s < 0.89, *p* > 0.43. The main effect of time was significant for FS, *F* (6, 2.46) = 27.68, *p* < 0.001, η_p_^2^ = 0.42, and FAS, *F* (6, 2.74) = 3.76, *p* = 0.016, η_p_^2^ = 0.09. Post hoc *t* tests with Bonferroni corrections (α′ = 0.008) were conducted to examine the differences across the successive time periods. Feeling states became more negative from min 6 to 11, *t* (39) = 3.21, *p* = 0.003, min 11 to 16, *t* (39) = 4.38, *p* < 0.001, and there was a significant increase in positive feeling states from min 21 at the completion of the trial to 5 min post trial, *t* (39) = 8.24, *p* < 0.001. All other comparisons failed to reach significance using α-corrected values, all *t*s < 2.29 *p* > 0.027. Arousal increased from pre-trial to min 1, *t* (39) = 3.80, *p* < 0.001, with all other changes not reaching significance using α-protected values, all *t*s < 2.56, *p* > 0.014.

### 3.3. Psychological States following the Running Trial

**Figure 3:**
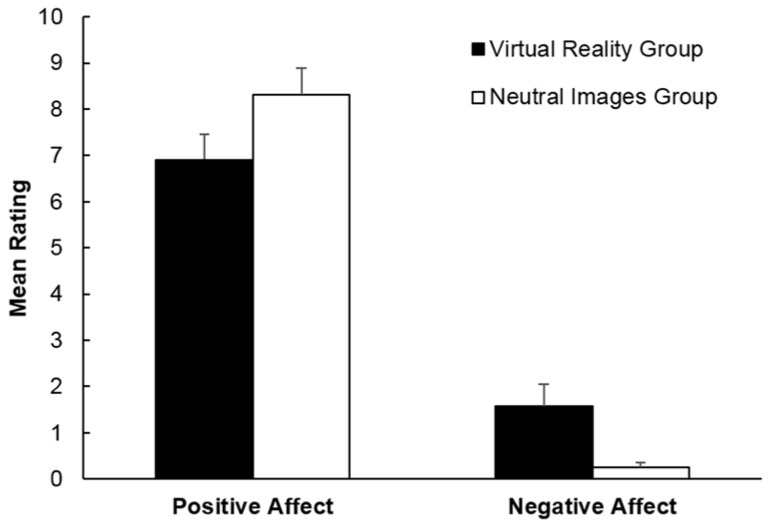
Mean positive affect and negative affect in the Virtual Reality group and Neutral Images group. Error bars depict the standard error of the mean.

Psychological states following the running trial were compared between groups separately for each outcome measure. As shown in Table 3, affect was generally more negative (or less positive) for the Virtual Reality group than for the Neutral Images group. Analyses showed that negative affect scores were significantly higher, *t* (25.59) = 2.78, *p* = 0.01, *d* = 0.74, and positive affect scores tended to be lower, *t* (38) = 1.69, *p* = 0.098, *d* = 0.55, in the Virtual Reality group than in the Neutral Images group. These differences between groups are shown in Figure 3. In addition, enjoyment scores were significantly higher for the Neutral Images group than for the Virtual Reality group, *t* (38) = 2.16, *p* = 0.037, *d* = 0.70. The differences between groups were not statistically significant for scores of fatigue, tranquillity, effort/importance, and pressure/tension, all *t*s < 1.33, *p* > 0.19. Due to the non-significant tendency for heart rate to differ between the groups, suggesting that physical exertion was higher in the Neutral Images group than in the Virtual Images group, further analyses were conducted using heart rate as a covariate. The analyses confirmed that the differences were significant for negative affect scores, *F* (1, 34) = 5.83, *p* = 0.021, η_p_^2^ = 0.15, positive affect scores, *F* (1, 34) = 5.12, *p* = 0.030, η_p_^2^ = 0.13, and enjoyment scores, *F* (1, 34) = 8.29, *p* = 0.007, η_p_^2^ = 0.20. In summary, the measurement of psychological states following the running trial indicated that affect was less negative, more positive, and enjoyment was higher in the Neutral Images group than in the Virtual Reality group.

### 3.4. Attentional Focus during the Running Trial

The percentage allocations to each of the categories in the MAF are shown in Table 4. Initial analyses were based on the two dimensional classification scheme of direction (internal, external) and relevance (association, dissociation) and used a 2 × 2 × 2 (Group × Direction × Relevance) ANOVA. As can be seen in Table 4, and supported by a main effect of direction, *F* (1, 38) = 192.74, *p* < 0.001, η_p_^2^ = 0.84, participants attended more to internal states and cues than to external states and cues. A main effect of association, *F* (1, 38) = 152.39, *p* < 0.001, η_p_^2^ = 0.80, also indicated that participants engaged in more associative focus than dissociative focus. However, both these main effects were qualified by a Direction × Association interaction, *F* (1, 38) = 134.48, *p* < 0.001, η_p_^2^ = 0.78. The interaction reflected that there was no difference between internal dissociative and external dissociative focus, *t* (39) = 0.19, *p* = 0.85, whereas there was greater internal associative focus than external associative focus, *t* (39) = 14.12, *p* < 0.001.

**Figure 4:**
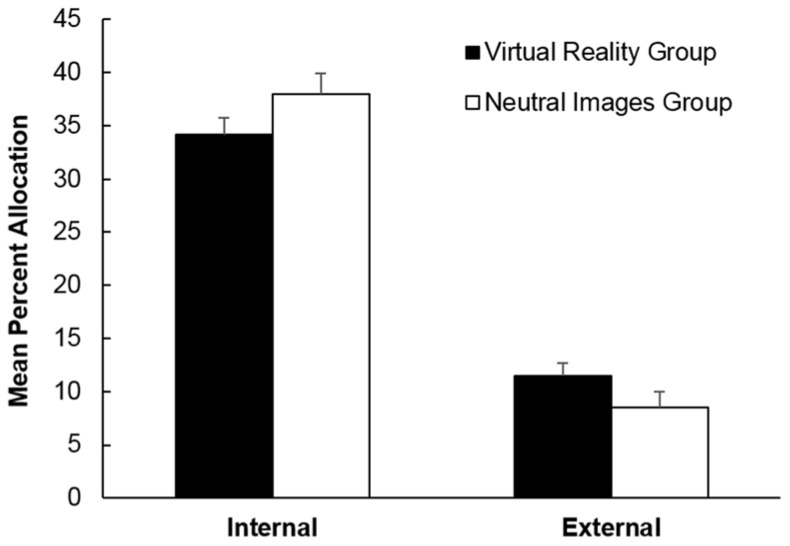
Mean percent allocation of time towards internal states and cues and external cues in the Virtual Reality group and Neutral Images group. Error bars depict the standard error of the mean.

The analyses also yielded a direction × group interaction that approached significance, *F* (1, 38) = 3.28, *p* = 0.078, η_p_^2^ = 0.08. The interaction is depicted in Figure 4. As can be seen, the Virtual Reality group engaged in more external focus of attention than the Neutral Images group. In contrast, there was less time allocated to attending internally in the Virtual Reality group than in the Neutral Images group. The ANOVA showed no further main effects or interactions, all *F*s < 1.

As noted, when attentional focus was associative, participants attended more towards internal cues and states than towards external cues. A 2 × 3 (Group × Type) ANOVA was conducted to examine the type of internal associative focus (bodily sensations, task-relevant thoughts, self-talk) further. The analyses yielded a main of Type, *F* (2, 76) = 7.23, *p* = 0.001, η_p_^2^ = 0.17. Follow up analyses using Bonferroni corrections (α′ = 0.017) showed that participants attended more to bodily sensations than to task-relevant thoughts, *t* (39) = 4.42, *p* < 0.001, and attended more to self-talk than to task-relevant thoughts, *t* (39) = 3.16, *p* = 0.003 (see Table 4). No significant difference was found between bodily sensations and self-talk, *t* (39) = 1.22, *p* = 0.23. The ANOVA yielded no main effect or interaction involving the group factor, *F*s < 1.

### 3.5. Relationships between the Measures for the Virtual Reality Group

Participants in the Virtual Reality group completed the ITQ to examine immersive tendencies that might be related to the effects of physical activity in the VR environment. Participants showed mean scores of 24.67 (*SD* = 3.36) for the Focus subscale, 23.42 (*SD* = 5.40) for the Involvement subscale, 17.87 (*SD* = 4.04) for the Emotion subscale, and 9.29 (*SD* = 4.34) for the Games subscale. Bivariate correlations were calculated to examine the relationships between individual differences in immersive tendencies and each of the dependent measures collected after the trial. The analyses showed that ITQ Focus subscale scores were positively correlated with the EFI Positive Affect subscale scores, *r* = 0.41, *p* = 0.049, and negatively correlated with the EFI Negative Affect subscale scores, *r* = −0.44, *p* = 0.03. In addition, the ITQ Emotion subscale scores were positively correlated with the IMI Pressure/Tension subscale scores, *r* = 0.51, *p* = 0.011, and the negative correlation with PACES enjoyment scores approached significance, *r* = −0.36, *p* = 0.081. Finally, the negative correlation between ITQ Involvement subscale scores and IMI Effort/Importance subscale scores approached significance, *r* = −0.36, *p* = 0.087. Taken together, the correlations suggest that a higher level of immersive tendencies was associated with more positive and less negative affective states following the VR running trial.

Participants in the Virtual Reality group also completed the RJPQ to examine responses to the VR environment. Participants gave mean scores of 31.96 (*SD* = 13.92) for the Reality Judgement subscale, 20.38 (*SD* = 7.91) for the Internal/External Correspondence subscale, and 6.92 (*SD* = 3.99) for the Attention/Absorption subscale. Significant correlations were also observed for the RJPQ and the measures of affect, enjoyment, and motivation. A significant negative correlation was found between Attention/Absorption subscale scores and both EFI Negative Affect subscale scores, *r* = −0.54, *p* = 0.007, and the IMI Pressure/Tension subscale scores, *r* = −0.57, *p* = 0.004. In addition, a significant positive correlation was found between Attention/Absorption subscale scores and PACES enjoyment scores, *r* = 0.44, *p* = 0.033. Similar to the ITQ, the correlations suggested that a higher level of attention/absorption in the virtual world was associated with more positive (or less negative) feeling states.

## 4. Discussion

The present study examined psychological states when engaging in physical exercise in a VR environment. Participants engaged in a 21 min run that increased from a light intensity to a vigorous intensity while either viewing the run in a VR environment that included other avatars, as well as their own, or while viewing neutral images. The present study is unique in that it used a control condition that involved the presentation of neutral visual stimuli rather than having participants engage in exercise in a “blank environment”. Few studies have also examined running in a virtual world or for a relatively long exercise duration that finished at a vigorous intensity level. The results revealed a similar pattern in perceived exertion and affect across the trial regardless of whether participants viewed a virtual world or neutral images. However, at the completion of the trial, participants who viewed the neutral images reported less negative affect and more positive affect and feeling states (e.g., enjoyment) than participants who completed the task in the virtual environment. In addition, participants who viewed the virtual environment tended to engage in more external-associative attentional focus than participants who viewed neutral images, whereas the opposite pattern was found for an internal-associative focus. Finally, analyses for the VR condition showed that a higher level of immersive tendencies and greater attention/absorption in the VR environment was associated with more positive (or less negative) feeling states.

The comparisons between the VR condition and the neutral images condition in the present study has resulted in significant insights into the nature of VR-based exercise. Neumann et al. [4] questioned the nature of the control condition used in previous research. It was pointed out that most control conditions involved performance of the physical activity on its own, without any feedback or distracting visual stimuli. For example, Murray et al. [7] compared rowing in a VR environment with a control condition that consisted of participants rowing in front of a blank wall. A blank environment control condition such as this may actually serve to increase the focus on negative bodily sensations associated with exercise [11]. The present study used a comparison condition in which participants viewed neutral images presented through the same apparatus as the VR environment. According to participant’s ratings on the MAF, this condition resulted in a focus of attention on bodily sensations for an average of about 20% of the time. The remaining time was spent on attending to other cues, including other internal-associative processes, such as self-talk and task-relevant thoughts.

Although running while viewing neutral images led to some focus of attention on bodily sensations, it resulted in less negative affect and more positive affect and enjoyment than running while viewing a virtual environment. This finding is somewhat surprising given the beneficial effects of VR-based exercise reported in previous research (see [4]). However, as previously noted, past research has used a blank environment control condition that does not control for participants receiving visual stimuli when exercising. The difference between conditions observed in the present experiment cannot be attributed to different levels of exertion. Perceived exertion and running speed did not differ significantly between conditions. There was a tendency for physical exertion as reflected in heart rate to be higher in the neutral images condition than in the VR condition. However, this cannot explain the present findings because higher physical exertion is typically associated with more negative and less positive affective states. Moreover, analyses using heart rate as a covariate confirmed that affect was more positive and less negative and that enjoyment was higher when participants viewed neutral images than when viewing a VR environment. 

One explanation for why affect was more positive and less negative in the when viewing neutral images than when viewing the VR environment is that it reflects different attentional processes across the two experimental conditions. As examined using the MAF, attention towards bodily sensations was overall higher in the VR condition than in the neutral images condition even though there was a tendency for a greater overall focus on internal-associative cues in the latter condition. The greater focus of attention on bodily sensations in the VR condition may have influence affective states, such that they were overall more negative and less positive than when viewing neutral images. 

An alternative explanation is that the VR condition lacked elements that induced a sufficient level of presence to yield strong enough psychological effects. The VR environment was shown as a monoscopic world through a projector onto the wall of the experimental room and this view remained fixed regardless of the participant’s head or eye movements. In addition, the view of the virtual world was from a first person perspective for only part of the trial because it was alternated between first and third person perspectives. A recent systematic review found that the features of stereoscopic vision, tracking level (e.g., use of head tracking), and a wide field of view had the largest effects on inducing a sense of presence in virtual environments [39]. As such, the VR condition used in the present study may have been limited in its capacity to induce presence. This, in turn, could have reduced the potential to observe affective benefits given the relationship between the level of presence and the strength of the psychological effects during physical exercise (e.g., [15]) and in other applications (see [3,39]).

In addition to assessing attention using the MAF, presence in the virtual environment was measured with the RJPQ. Importantly, it was only the subscale of attention/absorption that showed a relationship with affective states following the exercise task. The attention/absorption subscale was associated with reduced negative affect and pressure/tension and increased enjoyment together suggesting that greater attention to the virtual world improves the emotional experience of exercise. However, reality judgement and a sense of internal/external correspondence, which are also argued to be important elements of experiences in a virtual environment [35], were not related to affective states. The particular importance of attentional processes was supported by significant correlations observed between the ITQ Focus subscale and both positive and negative affect. The Focus subscale reflects the tendency to maintain focus in current activities. Thus, the ability to maintain focus in the virtual world may have facilitated the affective benefits of the VR-based running task.

Most researchers who have examined affective states have tended to measure these before and after, or only after, the VR task [5,11,14,15,16]. A handful of researchers have used both concurrent and post-task measures [7,13,40] as done in the present study. It is noteworthy that the differences between the VR and neutral images conditions in the present study were observed only for the post-task measures. A similar finding was reported by Murray et al. [7] in their comparison between rowing in a VR environment and rowing in a blank environment. The different results across the measurement periods may reflect that the concurrent measures reflect an assessment based primarily on momentary internal states that are evaluated relatively independent of external stimuli. In contrast, the post-task measures may elicit a more cognitive evaluation of the entire task, which includes a consideration of external stimuli.

Although the present study had a number of strengths, there are also limitations to note. A strength of the present study was that it employed a continuous exercise task of a relatively long duration (21 min). This allowed the tracking of perceived exertion and affect as exercise intensity increased from light to vigorous. It is also more similar to the types of exercise that individuals engage in and is aligned with physical activity guidelines. The long duration is also important if one argues that part of the benefit of VR is due to its novelty. The novelty and uniqueness of VR-based exercise might be expected to result in strong effects of VR for short duration exercise tasks but weaker effects for long duration exercise tasks because a longer duration will increase monotony and potentially result in boredom. The latter was attempted to be minimised in the present study by changing the view (first person versus third person) at regular intervals. Nevertheless, future research should examine the question of novelty, and its relationship with attentional engagement, by using tasks of varying duration or by observing changes over time when participants complete repeat sessions of VR-based exercise.

The present findings may be specific to the VR environment software and method to present the virtual environment. Similar to most other research (see [4]), the virtual environment was projected on to the wall of the experimental room. Although the image was large and somewhat immersive (as reflected in ratings on the RJPQ), the rendering of a 3D world on a 2D external screen is not the most immersive approach possible to render a virtual environment. The use of stereovision, wider field of view, and head tracking combined with relevant sounds from the virtual environment, may have produced a more immersive experience and stronger beneficial effects. Indeed, the present findings suggested that greater attention/absorption in the virtual environment was associated with more positive and less negative affective states. Nevertheless, the present methods had good ecological validity for the use of a VR environment when exercising on a treadmill. A HMD is not practical when running on a treadmill due to safety concerns [4]. In addition, a single flat screen is the most commonly used technology in commercial applications. As such, the present results highlight that using any VR technology is not always the most effective approach to improving affective states during exercise. To potentially produce more beneficial effects on affective outcomes, the VR exercise system may need to have technological features that increase presence and immersion (e.g., realistic rendering of the environment, a display that captures both focal and peripheral vision, multisensory stimulation) or has task features that increase engagement (e.g., competing against other avatars or runners, creating a game by having participants count passing landmarks or runners).

It may be argued that the experimental design may limit the conclusions that can be drawn. A third experimental condition of exercising in a blank environment was not used. The inclusion of this condition might have allowed greater comparisons with previous research. Based on the present results and those reported in past research (see [4]), it might have been expected that affect would be more negative when exercising in a blank environment than when exercising in a VR environment. Future research could include this additional control group to allow a more comprehensive examination of attention and affect when exercising in a VR environment.

In addition, the VR environment condition used in the present study include the presence of other avatars. As such, the VR condition differed from the neutral images condition in both the use of a virtual environment and the presence of other runners. The observed results in the VR condition may thus reflect one or a combination of these two factors. Nunes et al. [41] had participants run in a VR environment either alone or with an avatar that was either the participants own prior performance, a superior adversary, or an adversary chosen by the participant. Running with the avatar was found to be more motivating and result in higher perceived exertion and performance. However, in study by Nunes et al. [41] the conditions with the other avatar were framed as a competitor mode, whereas in the present study no such competition was implied. This difference is potentially important as having knowledge about being in competition with others can influence performance in VR-based exercise [41,42]. Moreover, different outcomes may result depending on whether the other avatar is perceived to be another competitor, a companion, or irrelevant. Nevertheless, future research could eliminate the potential impact of other avatars when making a comparison between exercise in a VR environment and an active control condition. In addition, the present study recruited participants that, while fit and generally active, were novice to exercising in a VR environment. It is not known what effects experience has in determining affective states during VR-based exercise. Thus, the present results may not apply to regular users of VR for exercise.

Finally, the present study assessed attentional focus using self-report measures. Future research might apply different approaches to measure attentional focus during the exercise task. The present method required participants to rate the percentage of time in which different type of foci were adopted. Although this method has been used in research to examine in naturalistic contexts (e.g., [43]) it may not fully capture attentional processes specific to interacting with virtual environments (e.g., internal/external correspondence of movements). It also relies on subjective reports, which can be prone to bias and requires the participant to have insight into their attentional states during the task. More objective measures of attention in VR environments could be used in future research, such as eye gaze [16], physiological measurements sensitive to attention (e.g., [44]), or secondary tasks (e.g., [45]). Alternatively, future research could examine attentional processes by manipulating focus of attention while participants exercise in a VR environment, as has been done with exercise tasks (e.g., [46,47,48]), and skill-based sports (e.g., [49]).

In conclusion, the present findings highlight the importance of attentional processes for affective and motivational responses while exercising in a VR environment. In particular, the capacity for the system to draw attention into the virtual world will increase the potential for higher levels positive affect and enjoyment. However, the current findings also suggest that VR applications may not always be the most effective approach to improving affective states during or following exercise. Simply presenting a changing visual stimulus can be equally or more effective in altering affect in a beneficial way. As such, it is important for future research to take into account the characteristics of the VR system (e.g., the capacity to engage attention), the individual (e.g., tendency to become immersed in virtual worlds), and the task (e.g., physical intensity level) when making conclusions about, or developing applications for, VR in enhancing exercise behaviour.

## Figures and Tables

**Table 1:** Demographic and physical activity variables for participants in the Virtual Reality group (*n* = 24) and the Neutral Images group (*n* = 16).

Variable	Virtual Reality Group	Neutral Images Group
Gender	Male = 11 Female = 13	Male = 8 Female = 8
Age (years)	*M* = 24.58 (range = 18–59)	*M* = 24.37 (range = 17–46)
BMI	*M* = 23.32 (*SD* = 3.67)	*M* = 22.63 (*SD* = 2.52)
IPAQ-SF Category	Low = 2 Medium = 5 High = 17	Low = 1 Medium = 4 High = 11
MET (min)	*M* = 4256.39 (*SD* = 5488.59)	*M* = 4585.09 (*SD* = 3987.82)
ETQ score	*M* = 57.46 (*SD* = 18.83)	*M* = 52.06 (*SD* = 15.47)

Note: Vmax = maximum velocity test; ETQ = Exercise Thoughts Questionnaire; IPAQ = International Physical Activity Questionnaire. MET = metabolic equivalent.

**Table 2:** Mean (standard deviations) ratings of perceived exertion (RPE) in the virtual reality group and neutral images group across the 21 min running trial.

Group	1 Min	6 Min	11 Min	16 Min	21 Min
Virtual Reality	9.13 (1.98)	11.39 (2.61)	12.87 (2.44)	13.91 (2.33)	15.09 (2.50)
Neutral Images	8.50 (2.03)	10.62 (1.82)	13.00 (1.93)	14.69 (2.52)	15.31 (3.59)

**Table 3:** Mean ratings (standard deviation) for psychological states measured following completion of the running trial in the Virtual Reality group and the Neutral Images group.

Measure	Virtual Reality Group	Neutral Images Group
Positive affect	6.92 (2.69)	8.31 (2.33)
Negative affect	1.58 (2.28)	0.25 (0.45)
Fatigue	5.00 (3.23)	3.75 (2.96)
Tranquillity	5.87 (2.17)	6.87 (3.03)
Enjoyment	58.67 (10.01)	64.94 (7.18)
Effort/Importance	28.04 (4.81)	27.00 (5.79)
Pressure/Tension	14.33 (5.27)	14.37 (5.67)

Notes: The Physical Activity Affect Scale was used to measure positive affect, negative affect, fatigue, and tranquillity; the Physical Activity Enjoyment Scale was used to measure enjoyment; and the Intrinsic Motivation Inventory was used to measure effort/importance and pressure/tension.

**Table 4:** Mean (standard deviations) for percentage of time allocated to different attentional focus types in the virtual reality group and neutral images group.

Attentional Focus Classification	Measure of Attentional Focus Category	Virtual Reality Group	Neutral Images Group
Internal association	Bodily sensations	28.21 (14.94)	20.25 (20.94)
	Task-relevant thoughts	11.50 (6.19)	14.50 (8.91)
	Self-talk	20.75 (16.27)	24.37 (17.11)
External association	Task-relevant external cues	13.75 (9.47)	9.12 (6.55)
Internal dissociation	Task-irrelevant thoughts	7.83 (7.26)	10.75 (10.81)
External dissociation	External distractions	9.21 (9.01)	7.81 (8.11)
